# Round Robin into Best Practices for the Determination of Indentation Size Effects

**DOI:** 10.3390/nano10010130

**Published:** 2020-01-10

**Authors:** Ana Ruiz-Moreno, Peter Hähner, Lukasz Kurpaska, Jacek Jagielski, Philippe Spätig, Michal Trebala, Simo-Pekka Hannula, Susana Merino, Gonzalo de Diego, Hygreeva Namburi, Ondrej Libera, Dimitry Terentyev, Tymofii Khvan, Cornelia Heintze, Nigel Jennett

**Affiliations:** 1 European Commission, DG-Joint Research Centre, Westerduinweg 3, 1755 LE Petten, The Netherlands; Peter.Haehner@ec.europa.eu; 2 National Centre for Nuclear Research, A. Sołtana str. 7, 05-400 Otwock–Świerk, Poland; lukasz.kurpaska@ncbj.gov.pl (L.K.); jacek.jagielski@ncbj.gov.pl (J.J.); 3 Laboratory for Nuclear Materials, Paul Scherrer Institute, 5232 Villigen, Switzerland; philippe.spatig@psi.ch; 4 Department of Chemistry and Materials Science, Aalto University, Kemistintie 1, 02150 Espoo, Finland; michal.trebala@aalto.fi (M.T.); simo-pekka.hannula@aalto.fi (S.-P.H.); 5 Centro de Investigaciones Energéticas, Medioambientales y Tecnológicas, (CIEMAT), Avda. Complutense 40, 28040 Madrid, Spain; susana.merino@ciemat.es (S.M.); g.diego@ciemat.es (G.d.D.); 6 Centrum Vyzkumu Rez, Hlavní 130, 250 68 Husinec-Řež, Czech Republic; hygreeva.namburi@cnl.ca (H.N.); ondrej.libera@cvrez.cz (O.L.); 7 Institute of Nuclear Materials Science, SCK-CEN, Belgian Nuclear Research Centre, Boeretang 200, 2400 Mol, Belgium; dterenty@sckcen.be (D.T.); tymofii.khvan@sckcen.be (T.K.); 8 Helmholtz-Zentrum Dresden-Rossendorf, Bautzner Landstraße 400, 01328 Dresden, Germany; c.heintze@hzdr.de; 9 Institute for Future Transport and Cities, Coventry University, Coventry CV1 5FB, UK; nigel.jennett@coventry.ac.uk

**Keywords:** nanoindentation, nano-mechanical, small scale testing, pile-up, elastic modulus correction, indentation size effect, ferritic/martensitic steel

## Abstract

The paper presents a statistical study of nanoindentation results obtained in seven European laboratories that have joined a round robin exercise to assess methods for the evaluation of indentation size effects. The study focuses on the characterization of ferritic/martensitic steels T91 and Eurofer97, envisaged as structural materials for nuclear fission and fusion applications, respectively. Depth-controlled single cycle measurements at various final indentation depths, force-controlled single cycle and force-controlled progressive multi-cycle measurements using Berkovich indenters at room temperature have been combined to calculate the indentation hardness and the elastic modulus as a function of depth applying the Oliver and Pharr method. Intra- and inter-laboratory variabilities have been evaluated. Elastic modulus corrections have been applied to the hardness data to compensate for materials related systematic errors, like pile-up behaviour, which is not accounted for by the Oliver and Pharr theory, and other sources of instrumental or methodological bias. The correction modifies the statistical hardness profiles and allows determining more reliable indentation size effects.

## 1. Introduction

Nanoindentation is extensively used to provide information about the mechanical behaviour of materials at the nano scale through the evaluation of force versus displacement curves measured during instrumented indentation. The most frequently used technique to calculate indentation hardness and moduli of materials is based on Oliver and Pharr’s method for the determination of the contact depth by accounting for the curvature of the unloading segment of the force-displacement data as described by a power law [1,2]. Recently, dynamic measurements in continuous stiffness measurements (CSM) mode have appeared where a small sinus oscillation is superimposed to the quasi-static load cycle and contact stiffness is continuously measured during loading. However, CSM methods present still some challenges to evaluate to what extent the measurement mode affects the results of the test. Indeed, a dependence of the derived mechanical properties on the oscillation parameters has been reported [3], as well as mismatches between static and dynamic indentation hardness due to strain rate sensitivities [4] and an influence of instrumental artefacts on the measured stiffness [5]. Beyond the measurement of mechanical properties at small scale, nanoindentation can also be used as an experimental tool for studying fundamental materials physics such as the formation of dislocation networks, plastic instabilities, and phase transformations [6]. Continuous refinement of experimental and modelling methodologies to assess mechanical properties at local level is enabling nanoindentation as a technique to obtain microstructural information [7,8,9,10,11], notably through the exploitation of indentation size effects (ISE) [12,13,14,15], which represents a link between small-scale mechanical and microstructural properties. However, the usefulness of the extracted information depends on the reliability of nanoindentation measurements. Common uncertainties in nanoindentation measurements cause bias and scatter of the measured values and originate from the key calibrations of the instrument (Displacement, Force, Indenter area function, Frame compliance), the zero-point determination to establish the initial depth of penetration, and from the random noise contributions from the environment such as ground and acoustic vibrations causing variation in force and displacement measurement. The results obtained are also affected by the models used for the evaluation of data and the data analysis corrections for variation in the exponent of the power law fit to the data and lateral dilation correction of the indentation contact [16]. Specimen roughness can affect both the zero point and the actual area of contact and residual stress (which may be intrinsic to the sample manufacturing route or polishing-induced) causes an error in the contact mechanics estimate of the actual contact area. Joslin and Oliver [17] have presented a method to remove the errors due to surface roughness by analyzing the composite parameter hardness/modulus^2^ (*H*/*E*^2^) instead of treating hardness and modulus separately. Other potential errors include surface forces/adhesion and material exhibiting pile-up or sink-in behaviour, which can vary across the same sample, depending on the ratio of local yield stress (and so hardness) to elastic modulus and the orientation of the indenter geometry to the local crystal orientation. Awareness about the possible influences and errors in nanoindentation measurements is critical to elaborate practices and methodologies that eliminate or reduce them or take them into account. A more detailed consideration regarding estimation of uncertainties in instrumented indentation can be found in [18] and in ISO 14577:2015-Annex H. Once valid indentation data have been obtained, there remains the issue of indentation size effects, where smaller indentations are harder because the yield stress of materials that deform via dislocation generation and movement has a fundamental length-scale dependence [7,8,19,20].

T91 and Eurofer 97 are tempered martensitic steels which are candidate materials for structural components in nuclear fission and fusion reactors. To predict the long-term material behaviour at operating conditions in nuclear environments, their deformation behaviour under high irradiation dose levels must be characterized. Nanoindentation and other more recent micromechanical testing approaches have proved promising to assess radiation damage, either caused by ions or by neutrons, thanks to the possibility of testing shallow depths affected by ion irradiation and/or small volumes of activated materials after neutron irradiation [21,22,23]. Due to the limited availability of irradiated samples, developing methodologies for robust characterizations as performed in different laboratories and by different instruments is advantageous. The methodology should take into account pile-up formation, since the ferritic/martensitic steels exhibit high dislocation density and therefore, significant resistance to dislocation motion and low strain hardening capability, which may force the material upwards during the indentation process [16]. The Oliver and Pharr procedure [1] will then produce inaccurate results because it does not take modifications of the contact area due to pile-up behaviour into account [24]. In line with the original work by Joslin and Oliver [17], it has been recently demonstrated that uncertainties related to the contact area determination (pile-up and residual stresses) can be compensated during data analysis if the elastic properties of the material are a priori known and a correction factor can be applied to the hardness values [23,25,26].

In this framework, seven laboratories have engaged in a round robin testing campaign to probe T91 and Eurofer97 surfaces prepared by the same sample preparation method and using the same measurement protocols in various quasi-static nanoindentation testing modes. This work presents an analysis of the nanoindentation data obtained by the different devices, accounting for uncertainties in the contact area, in order to define a best practice methodology for the determination of indentation size effects.

## 2. Materials and Methods

### 2.1. Materials

Two ferritic/martensitic steels, namely T91 and Eurofer97, were used for this study. The chemical composition of the steels is given in Table 1. T91 specimens were cut from a hot rolled plate normalized at 1050 °C during 1 min/mm (per mm thickness), quenched to room temperature, tempered at 770 °C for 3 min/mm and then cooled in air. Eurofer97 samples were cut from broken Charpy specimens prepared from forged bars hardened at 979 °C for 1 h 51 min and tempered at 739 °C for 3 h 42 min. The materials and their tempering treatment were chosen for their featuring a nanoscopic martensite lath structure with characteristic lath sizes in the range of 100 to 200 nm [13] that is fine enough to have nanoindentation probe an effective medium thereby minimizing the effect of grain structure and crystallographic orientation on hardness measured.

**Figure 1:**
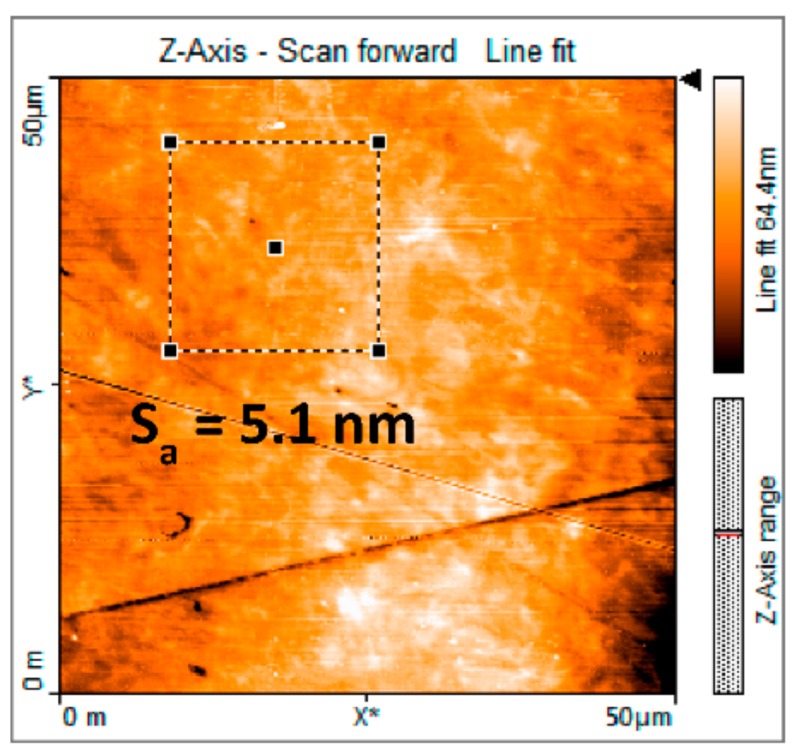
50 × 50 µm^2^ AFM scan of Eurofer97 showing a surface area roughness of 5.1 nm.

The materials were cut into plates of 1 mm thickness and polished with successively finer abrasives and polishing solutions (diamond suspensions of 9 µm, 3 µm and 1 µm particles) finalising by a gentle manual polishing in oxide polishing suspension with silica nanoparticles for 5 min. All samples were polished in one laboratory and distributed to the laboratories participating in the study. The roughness of the surface was checked by Atomic Force microscopy to be below 20 nm (Figure 1). The surface residual stresses were checked in one sample of each material using X-ray diffractometry. The values in both materials were close to 425 MPa compressive stress.

### 2.2. Nanoindentation Tests

Indentation tests were conducted in a variety of nanoindentation test devices from several providers (Anton Paar, Corcelles, Switzerland; Bruker, Santa Barbara, CA, USA; Micro Materials Ltd., Wrexham, UK; MTS, Eden Prairie, MN, USA; Keysight (former Agilent), Santa Rosa, CA, USA; Zwick-Roell, Ulm, Germany). The thermal drift at room temperature was below 0.05 nm/s for the different nanoindentation systems used in this study. The maximum load of the devices varied from 10 mN to 10 N, the load resolution from 1 nN to 100 nN and the displacement electronic resolution from 0.3 pm to 50 pm. However, no distinction about the instruments’ capabilities is made in the aggregation of data and statistical analyses that follow.

All tests were conducted at room temperature using Berkovich diamond tips. The tip area function and the instrument frame compliance were calibrated according to ISO 14577-2:2015. Three quasi-static nanoindentation measurement modes have been applied for the comparison of ISE, namely force controlled single cycles (FSC), depth controlled single cycles (DSC) and progressive multi-cycles in force control (PMC). FSC measurements were performed at five maximum forces, *F*_max_, equal to 1 mN, 5 mN, 10 mN, 50 mN and 100 mN, using 30 s of loading and unloading ramp times and 10 s dwelling at *F*_max_. The instruments with maximum load below 100 mN performed the measurements at the *F*_max_ values divided by 10. DSC measurements were performed up to maximum depths, *h*_max_, ranging from 50 nm to 500 nm, using 30 s of loading and unloading ramp times and 10 s dwelling at *h*_max_. PMC measurements were performed applying 10 consecutive loading-unloading cycles with the force being increased by 0.1·*F*_max_ in each cycle from 10 mN to 100 mN (or from 1 mN to 10 mN for the systems reaching maximum loads below 100 mN). In every cycle the loading, unloading and dwelling times were set to 10 s and the unloading was conducted down to 30% of the maximum force of the cycle. For drift correction, a final 60 s dwelling at 10% of *F*_max_ or *h*_max_ was applied to all measurements prior to complete unloading. At least 15 measurements were taken and averaged for each *F*_max_ in FSC and for each *h*_max_ in DSC. As well, 15 PMC measurements of 10 cycles were taken and the results of the 15 measurements were averaged cycle by cycle.

### 2.3. Data Analysis

The data were analysed according to ISO 14577-1:2015 and using Oliver & Pharr methodology [1] after application of zero point and thermal drift corrections. Contact depth *h*_c_, indentation hardness *H*_IT_, and reduced plane strain modulus of the contact *E*_r_, are determined by Equations (1)–(3):

(1) $$h_{c}=h_{\max}-\varepsilon \left(h_{\max}-h_{r}\right)$$

(2) $$H_{\mathrm{IT}}=\frac{F_{\max}}{A_{p}\left(h_{c}\right)}$$

(3) $$E_{r}=\frac{\sqrt{\pi }S}{2\beta \sqrt{A_{p}\left(h_{c}\right)}}$$

where *h*_max_ is the depth at maximum force, *h*_r_ is the tangent depth, *ε* is a correction factor dependent on the indenter geometry and the extent of plastic yield in the contact (0.6 < *ε* < 0.8), *A*_p_ is the projected area of contact, *S* is the stiffness, and *β* is a geometric factor set to 1.034 for a Berkovich indenter. *β* is introduced to correct the analysis equations, based on the geometry of an axis-symmetric cone, to the shape of a Berkovich indenter [27].

## 3. Results

### 3.1. Hardness and Modulus Profiles

All samples were prepared by one laboratory while the samples were then tested at different locations by methods agreed in advance and in controlled environments with stable levels of temperature and humidity. Therefore, the test results are not expected to depend primarily on the laboratory environment, the ageing of the materials, surface oxide formation or any other time-dependent response, assuming a proper correction for thermal drift has been made. All measurements have an inherent variability due to the random uncertainties of the test method. Between different laboratories and instruments, additional offsets are possible. Typical sources of variability and offsets between different laboratories are due to:

- calibration differences (force, displacement, the calibration of the indenter area function and the correction of the frame compliance) and other measurement uncertainties,
- sample to sample property variations (compositional variation, polishing differences, residual stress, etc.),
- or could be due to differences in the details of the analysis methods applied in the software of each instrument: Oliver and Pharr uses a beta factor of 1.034, whereas ISO 14577:2002 uses a factor of 1; software compliant with ISO 14577:2015 applies a variable ε (i.e., determines a correction factor for ε which depends on the exponent of the power law fitting the unloading curve) and a lateral dilation correction to the contact area calculation (which depends on the hardness to elastic modulus ratio of the test piece) [28].

**Figure 2:**
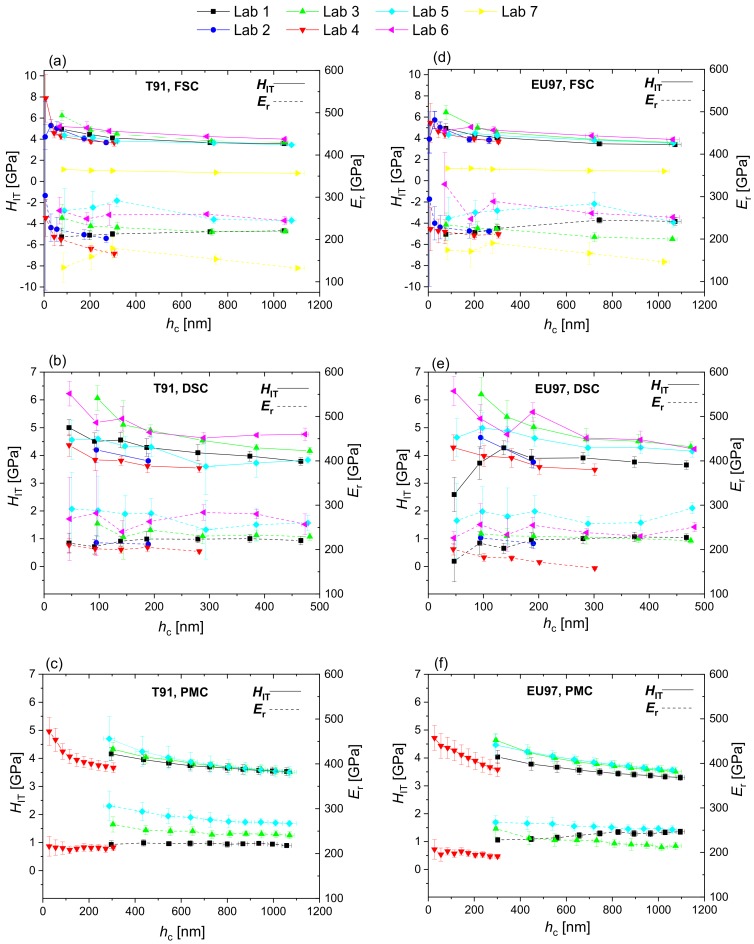
Indentation hardness, *H*_IT_, and reduced modulus, *E*_r_, of T91 (**a**–**c**) and Eurofer97 (**d**–**f**) measured by single cycles in force control (FSC), single cycles in depth control (DSC) and multicycles in force control (PMC) at different laboratories.

**Figure 3:**
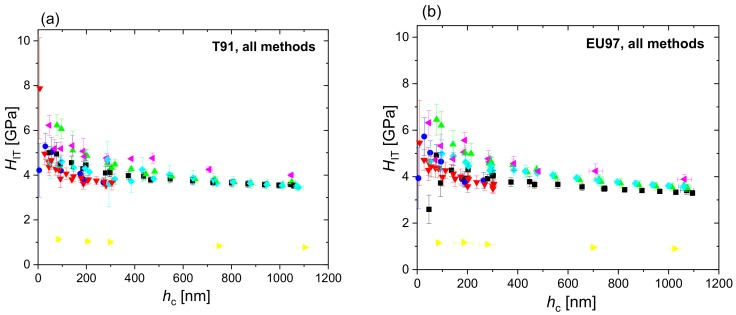
Indentation hardness, *H*_IT_, of T91 (**a**) and Eurofer97 (**b**) measured at the different laboratories for all methods combined (FSC, DSC and PMC).

Figure 2 shows the hardness and reduced modulus of T91 and Eurofer97 as a function of contact depth measured in the different laboratories using FSC, DSC and PMC nanoindentations, while Figure 3 shows the hardness profiles combined for all measurement methods and laboratories. The datasets used to plot Figure 2 and Figure 3 are provided as Supplementary Materials (see Spreadsheet S1). The hardness profiles (Figure 2) exhibited comparatively low scatter for FSC and PMC methods, whereas a large scatter was observed in the modulus values. The calculation of hardness depends on the calibration of the projected area and the determination of the contact depth. In addition to these factors, the calculation of the modulus depends also on the determination of stiffness. The prevalence of scatter in the modulus values indicates that there is a higher uncertainty in the determination of stiffness by the different testing devices than in determining contact depth. The extreme outlier observed in Figure 3 (Lab 7, yellow data) is probably due to an inaccurate instrument calibration, in particular a largely underestimated frame compliance.

### 3.2. Elastic Modulus Correction (EMC)

T91 and Eurofer97 are relatively high-strength and low-strain hardening materials, with a moderate modulus to hardness ratio (*E*/*H*) in the order of 60. Because of these properties, one may suspect the formation of pile-ups during indentation. Indeed, the values of *h*_p_/*h*_max_ are about 0.9, well beyond the 0.7 threshold above which pile-up occurs [2,19]. Effects of pile-up are mainly reflected in a systematic error in the determination of the projected contact area and, thus, an overestimation of hardness and modulus. To account for these effects, an elastic modulus correction [25,26] has been applied to the hardness values whereby hardness is corrected by a factor depending on the ratio of the measured reduced modulus to a reference reduced modulus value, assuming that the elastic modulus is independent of depth. The reference value for the reduced modulus, *E*_r_^ref^, has been calculated by Equation (4):

(4) $$\frac{1}{E_{r}^{\mathrm{ref}}}=\frac{1-\nu_{i}^{2}}{E_{i}}+\frac{1-\nu_{s}^{2}}{E_{s}^{\mathrm{ref}}}$$

where *υ_s_* is the Poisson ratio of the steel samples set to 0.3, *υ_i_* is the Poisson ratio of the diamond indenter set to 0.07, *E_i_* is the indenter modulus set to 1141 GPa, and *E*_s_^ref^ is the macroscopic elastic modulus of the steel samples, in this study set to 208 GPa for T91 and 217 GPa for Eurofer97. Applying Equation (4), the reference reduced moduli for T91 and Eurofer97 are 190.6 GPa and 197.4 GPa respectively.

(5) $$H_{\mathrm{IT},\mathrm{corr}}=\frac{H_{\mathrm{IT}}}{{\left(\frac{E_{r}\left(h_{c}\right)}{E_{r}^{\mathrm{ref}}}\right)}^{2}}$$

We note that EMC is equivalent to analyzing *H*_IT_/*E*_r_^2^, or according to Equations (2) and (3), *F*_max_/*S*^2^ which was applied by Joslin and Oliver in order to remove the errors due to surface roughness [17]. As these authors noted *H_IT_*/*E_r_*^2^ is a better indication of the material’s resistance to permanent indentation than hardness or modulus alone.

**Figure 4:**
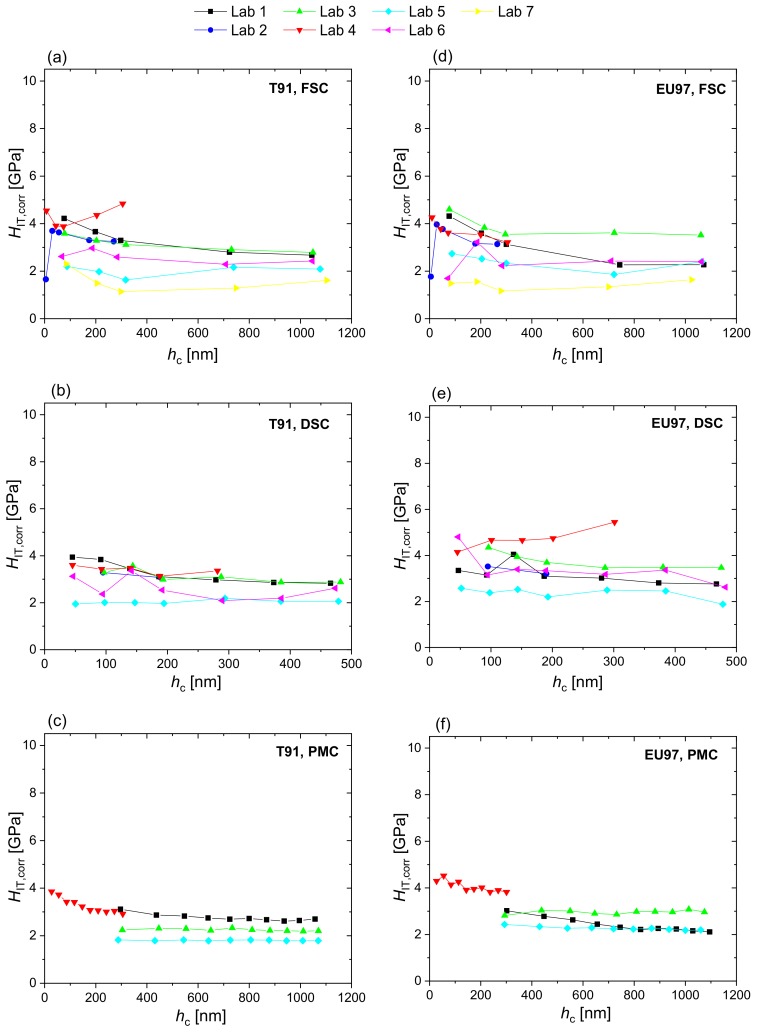
Indentation hardness corrected by the EMC factor, *H*_IT,corr_, of T91 (**a**–**c**) and Eurofer97 (**d**–**f**) measured by single cycles in force control (FSC), single cycles in depth control (DSC) and multicycles in force control (PMC) at different laboratories.

**Figure 5:**
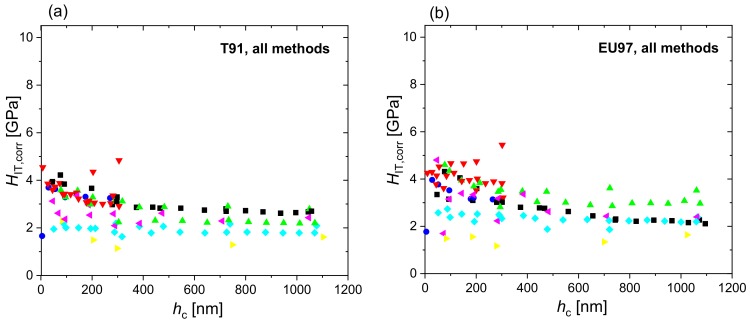
Indentation hardness corrected by the EMC factor, *H*_IT,corr_, of T91 (**a**) and Eurofer97 (**b**) for all methods combined (FSC, DSC and PMC).

Figure 4 shows the corrected hardness profiles of T91 and Eurofer97 measured in the different laboratories using FSC, DSC and PMC nanoindentation, while Figure 5 shows the corrected hardness profiles combined for all measurement methods and laboratories. EMC provides a correction of individual *H*_IT_ values. Comparing Figure 3 and Figure 5 it is evident that EMC increases the scatter in the results of each laboratory, while it decreases inter-laboratory sources of scatter and helps to identify outliers (e.g., Lab 4, Lab 5 and Lab 7 in Figure 4a, or Lab 5 and Lab 6 in Figure 4b). The latter improves data quality and comparability as it could effectively correct for calibration offsets between laboratories, as can be seen by the fact that the extreme outlier observed in the raw indentation hardness profiles (yellow data, Lab 7 in Figure 3) get much closer to the curves obtained in the rest of laboratories after EMC is applied (Figure 5). EMC also reduces indentation size effects by correcting the overestimation in hardness due to pile-ups.

### 3.3. Cross-Correlation Analysis before and after EMC

A statistical study has been carried out to investigate which combination of measurement method and analytical correction works better to minimise intra- and inter-laboratory variations. A number of mathematical functions have been proposed in the literature to describe ISE. Their basis has ranged from empirical (Hall–Petch relation [29,30]) through to physical based arguments such as strain gradient plasticity (Nix–Gao model [31]) and slip distance theory (Hou–Jennett model [7]). One simple function is an exponential function. For the current statistical study, the raw data and the elastic modulus-corrected hardness profiles have been fitted to exponential functions (Equation (6)), serving as reference for analysing standard deviations in depth-dependent hardness and evaluating the quality of data:

(6) HITfit=H0+H1·e−hch1

The choice of the exponential functions is motivated by the fact that this class of functions offers sufficient variability to describe the decay of hardness with increasing depths while getting along with a minimum number of (three) fit parameters. Hence, the choice represents a matter of practicality for quantitatively analyzing the combined inter- and intra-laboratory scatter in terms of the goodness of fits, not a choice made on physical grounds in order to determine an ISE accurately.

**Figure 6:**
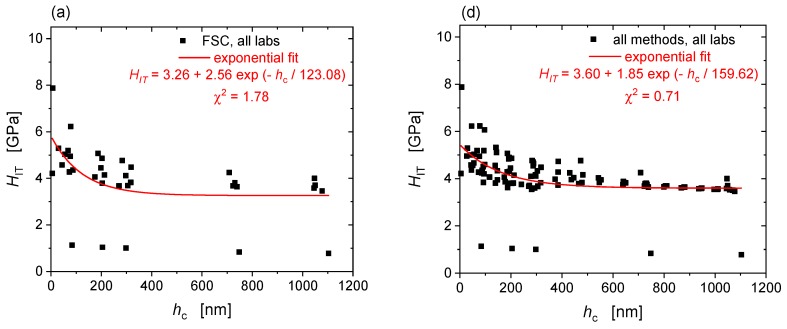

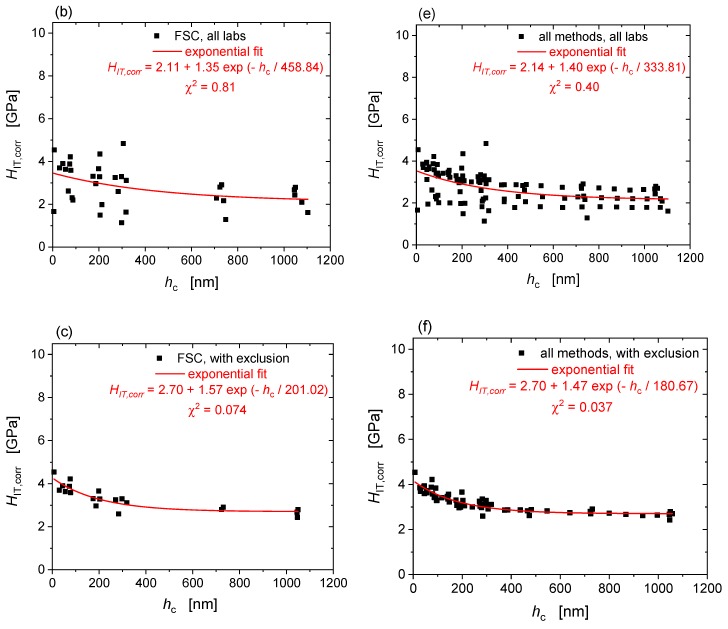
Exponential fits to raw hardness data, EMC corrected hardness and EMC corrected hardness with exclusions of T91 measured in FSC mode (**a**–**c**) and by all methods combined (**d**–**f**).

The EMC hardness plots (Figure 4 and Figure 5) allow outliers to be identified and excluded from the characterization. The corrected hardness with exclusions has also been fitted to exponential curves and statistically analysed in terms of the deviation between measured and expected values (goodness-of-fit) as well as of degree of correlation with the measured elastic properties (cross-correlations of hardness and reduced modulus). Figure 6 shows the exponential fits to the raw hardness data, the EMC corrected hardness and the EMC corrected hardness with exclusions of measurements in FSC mode (Figure 6a–c) and measured by all methods (Figure 6d–f) for T91. The same analysis has been done for the sets of data from DSC and PMC measurement modes as well as for Eurofer97 (plots not shown). 

The goodness of the fits has been evaluated by the standard error of the regression (reduced Chi-squared, χ^2^) and used to assess the intra-laboratory data scatter. Inter-laboratory deviations have been assessed by the standard deviation, σ^R^, of the cross-correlation functions of hardness and reduced modulus given by Equations (7) and (8) for raw hardness and EMC corrected hardness respectively:

(7) $$R_{H_{\mathrm{IT}}E_{r}}=\frac{\langle \left(H_{\mathrm{IT}}-H_{\mathrm{IT}}^{\mathrm{fit}}\right)\cdot \left(E_{r}-E_{r}^{\mathrm{ref}}\right)\rangle }{\sigma_{H_{\mathrm{IT}}}\cdot \sigma_{E_{r}}}$$

(8) $$R_{H_{\mathrm{IT},\mathrm{corr}}E_{r}}=\frac{\langle \left(H_{\mathrm{IT},\mathrm{corr}}-H_{\mathrm{IT},\mathrm{corr}}^{\mathrm{fit}}\right)\cdot \left(E_{r}-E_{r}^{\mathrm{ref}}\right)\rangle }{\sigma_{H_{\mathrm{IT},\mathrm{corr}}}\cdot \sigma_{E_{r}}}$$

Table 2 lists the *χ*^2^ values representing the goodness of the fits and the standard deviation of the hardness vs. modulus cross-correlations for FSC, DSC, PMC and all-methods data sets obtained for T91 and Eurofer97. In the case of the auto-correlations, in general the deviation decreases when the EMC correction is applied, except for the PMC method, which already presented a very small *χ*^2^ in the raw data (*χ*^2^ = 0.05). Applying the exclusions, the *χ*^2^ values drastically decrease in all cases, the lower values being achieved by using the PMC method, both in T91 and in Eurofer97. Regarding cross-correlations, the EMC hardness presented more discrepancy as correlated to the reduced modulus (higher standard deviation of the hardness-reduced modulus cross-correlation function), while it significantly decreases by applying exclusions. Again, the PMC method presented the highest cross-correlation.

## 4. Discussion

### 4.1. Uncertainty Analysis and Effects of Elastic Modulus Correction

Accuracy achieved in deriving mechanical properties from nanoindentation measurements is affected by both random errors and systematic bias occurring either during the testing procedure or in the data analysis phase. Random errors, e.g., indenting on a pit in the sample surface, are difficult to correct, but can often be reduced by averaging many results; systematic error can appear as a measurement bias, which can be estimated and corrected for. The origins of systematic errors include laboratory-specific errors, such as inaccurate calibrations of force, displacement, frame compliance and indenter tip shape, or material/sample-specific factors, such as surface and bulk residual stresses and pile-up or sink-in behaviour during indentation. The different sources of systematic error produce different effects in the measured indentation hardness and modulus. A blunt indenter tip calibrated correctly will produce the same modulus but a different hardness to a sharp tip. Compressive residual stresses (e.g., due to mechanical polishing) and pile-up behaviour produce an apparent increase in both hardness and modulus, whereas a too small frame compliance correction reduces modulus and hardness. A high surface roughness would tend to cause random uncertainty. It can cause a variable offset of the zero point from the average surface position of the sample and would cause a variation in the actual area of contact with the indenter. Depending on its lateral surface wavelength, roughness may cause increase or decrease in measured stiffness. When the indent size is smaller than the roughness wavelength, this results in indentation in hills or valleys, causing an offset in the contact depth and or stiffness measured due to local curvature of the sample surface. When the indent size is much greater than the roughness wavelength, the contact senses a less dense/low modulus surface layer of asperities before the onset of contact with fully dense material. Roughness, therefore, produces a complex series of conflicting effects on hardness and modulus parameters extracted from a nanoindentation test and this is why the standard ISO 14577 restricts indentation into surfaces to be where average roughness is less than 5% of the contact depth.

The elastic modulus correction applied in this study relies upon the assumption that there is no error in the measured stiffness. If this assumption holds, EMC reduces the error that compressive residual stresses and pile-up behaviour cause in the estimate of the area of contact. However, if an imprecise frame compliance correction has been applied, this is an error in the stiffness value measured and not in the area of contact; in this case the specific correction formula used will introduce a compensating error rather than a correction. The use of elastic modulus of a reference material to obtain the value of frame compliance in a nanoindenter is a standard procedure (see ISO 14577-2:2015) but this relies upon the opposite assumption, i.e., that the area of contact obeys the assumptions of the contact mechanics analysis being applied to calculate the contact stiffness and that the error is in the contact stiffness alone. While the raw hardness profiles of individual datasets (Figure 2 and Figure 3) are consistent with a monotonic decrease of hardness with depth (ISE), there is a large variability amongst the different laboratories regarding hardness (ISE) and modulus profiles. In stiff indents, such as at high force or in material with high modulus-to-hardness ratio ($\frac{E}{\sqrt{H}}$), the force removal curve is very steep and small errors in frame compliance, force or displacement (e.g., due to vibrations, drift or creep) can cause large changes in the measured stiffness and result in a high uncertainty in *E*. However, the estimate of contact depth is little affected and so the uncertainty in *H* is low. The result is a large measurement variability in *E* and a low measurement variability in *H*. In this case, EMC generates a correction factor that compensates for and nulls out the random uncertainty in the modulus results. When this is applied to the hardness values, it significantly increases the scatter of data in the hardness profiles (Figure 4 and Figure 5). This increase in random uncertainty can, however, be reduced or avoided by using averaged results. The standard error of an averaged stiffness measurement, even with large random uncertainty, is rapidly reduced by averaging a greater number of measurement results, which becomes easily possible by using the PMC method. Even though the in-house scatter of hardness results increased after EMC, in this case, the approach was necessary to account for the systematic offsets in the indentation results caused by pile-up and residual stresses that exist in both materials. Furthermore, changes of pile-up behaviour caused by irradiation, if not corrected for, can lead to ambiguous results when using nanoindentation to study irradiation induced hardening of materials of nuclear interest. Reduction of offsets between data sets through EMC, normalises the data into a single statistical population that can be used as a base to identify outliers, the exclusion of which largely reduces the overall variability, as revealed by the example statistical analysis performed (Figure 6 and Table 2) and discussed below.

### 4.2. Statistical Analysis of Measurement Methods for Improved ISE Determination

The nanoindentation response of the two ferritic/martensitic steels resemble each other and so do the outcomes of the statistical analysis. In both cases, amongst the different testing methods PMC outperformed the two other methods in terms of goodness of fits, to the extent that already the regressions of the raw PMC datasets are better than the corrected FSC and DSC datasets. Likewise, the residual cross-correlations provide evidence that PMC performs much better than the single cycle methods. This is likely because in PMC, the depth dependence of hardness and modulus are measured at the same point on the surface of the specimen, while FSC and DSC probe different points of the surface to measure the hardness and modulus profiles. Thus, random errors introduced from point to point sample variability (such as surface inhomogeneity) will affect more the single cycle measurements and this may be reflected in the larger scatter and poorer cross-correlation of single cycle modes. In addition, the results for different depths in the case of the PMC method rely on the same zero-point determination, thus reducing the scatter related to uncertainties related to zero-point correction. Also contributing is the fact that more data points were obtained when using PMC (data was obtained at 10 depths because 10 cycles were applied, providing 10 averaged data points in the hardness and modulus profiles) as compared to FSC (5 data points in the hardness and modulus profiles) and DSC (7 data points). The standard error in a fit is reduced as the number of fitted data points increases.

For both materials, EMC hardness compilations where outliers have been removed exhibit significantly improved goodness of the exponential fits to the depth dependent hardness data as well as the residual cross-correlations between hardness and modulus. Therefore, the EMC offers a strong approach towards obtaining reliable hardness profiles to study and exploit ISE, in particular for materials amenable to pile-up or sink-in behaviour during the indentation process.

## 5. Conclusions

Nanoindentation has been widely used for qualitative purposes, e.g., comparative screening of materials. Special attention has to paid to proper calibration of: force, displacement, frame compliance and indenter tip area; to a precision that would allow for quantitative hardness and elastic modulus determination. In particular, calibration of the frame compliance and the tip area function are critical for the present inter-laboratory comparison. While systematic errors associated with the correction of frame compliance are still to be considered, systematic errors originating from the projected contact area determination (tip area calibration, pile-ups and residual stresses) have been taken into consideration and significantly reduced by the application of an Elastic Modulus Correction, as evidenced by the statistical examination of hardness profiles showing improved goodness-of-fits and hardness-to-modulus cross-correlation when EMC is applied and used to identify outliers. The methodology provides a robust framework for the study of size dependent mechanisms of deformation based on nanoindentation testing.

## Figures and Tables

**Table 1:** Chemical composition of the T91 and Eurofer97 materials (in wt.%; Fe balance).

**Element**	**Cr**	**Mo**	**Mn**	**Si**	**V**	**Ni**	**Nb**	**Cu**	**Al**
**T91**	8.873	0.871	0.386	0.218	0.195	0.115	0.077	0.080	0.009
**Eurofer97**	8.87	<0.001	0.42	0.06	0.19	0.0075	<0.001	0.021	0.008
**Element**	**C**	**N**	**P**	**S**	**Sn**	**O**	**W**	**Ta**	**Ti**
**T91**	0.097	0.0440	0.020	0.0005	-	-	-	-	-
**Eurofer97**	0.12	0.018	0.004	0.003	<0.005	0.001	1.10	0.14	0.008
**Element**	**Co**	**As**	**Sb**	**Zr**					
**T91**	-	-	-	-					
**Eurofer97**	0.005	<0.005	<0.005	<0.005					

**Table 2:** Standard error of the exponential fit regressions to raw hardness data and EMC corrected hardness, *χ*^2^, and standard deviation of cross-correlations between hardness and elastic modulus, σ^R^, for T91 and Eurofer97 obtained from indentations using different control measurement modes.

Material	Method	Goodness of Hardness Fits, *χ*^2^			Standard Deviation of Cross-Correlations, σ^R^		
		Raw, *H*_IT_	EMC, *H*_IT,corr_	EMC with Exclusion, *H*_IT,corr_	Raw, *H*_IT_	EMC, *H*_IT,corr_	EMC with Exclusion, *H*_IT,corr_
T91	FSC	1.78	0.81	0.074	0.96	1.28	0.498
	DSC	0.35	0.31	0.029	1.67	2.25	0.589
	PMC	0.05	0.14	0.005	1.50	1.64	0.187
	All methods	0.71	0.40	0.037	1.26	1.61	0.487
EU97	FSC	1.58	0.81	0.159	0.75	1.40	0.797
	DSC	0.57	0.69	0.144	0.85	1.43	0.785
	PMC	0.05	0.15	0.020	1.03	0.81	0.254
	All methods	0.71	0.56	0.143	0.87	1.49	0.759

## Supplementary Materials

The following are available online at https://www.mdpi.com/2079-4991/10/1/130/s1, Spreadsheet S1: Datasets of hardness, reduced modulus and contact depth values of T91 and Eurofer97 measured by quasi-static nanoindentation in different laboratories.

## Abbreviations

CSM	Continuous Stiffness Measurements
DSC	Depth Controlled Single cycles
EMC	Elastic Modulus Correction
FSC	Force Controlled Single cycles
ISE	Indentation Size Effects
ISO	International Standard Organization
PMC	Progressive Multi-Cycles in force control

## References

[1] Oliver W.C., Pharr G.M. (1992). An improved technique for determining hardness and elastic modulus using load and displacement sensing indentation experiments. J. Mater. Res..

[2] Oliver W.C., Pharr G.M. (2004). Measurement of hardness and elastic modulus by instrumented indentation: Advances in understanding and refinements to methodology. J. Mater. Sci..

[3] Siu K.W., Ngan A.H.W. (2013). Oscillation-induced softening in copper and molybdenum form nano- to micro-length scales. Mater. Sci. Eng. A.

[4] Leitner A., Maier-Kiener V., Kiener D. (2017). Dynamic nanoindentation testing: Is there an influence on a material’s hardness?. Mater. Res. Lett..

[5] Merle B., Maier-Kiener V., Pharr G.M. (2017). Influence of modulus-to-hardness ratio and harmonic parameters on continuous stiffness measurement during nanoindentation. Acta Mater..

[6] Schuh C.A. (2006). Nanoindentation studies of materials. Mater. Today.

[7] Hou X., Jennett N.M. (2012). Application of a modified slip-distance theory to the indentation of single-crystal and polycrystalline copper to model the interactions between indentation size and structure size effects. Acta Mater..

[8] Hou X.D., Bushby A.J., Jennett N.M. (2008). Study of the interaction between the indentation size effect and Hall-Petch effect with spherical indenters on annealed polycrystalline copper. J. Phys. D Appl. Phys..

[9] Zhao M., Slaughter W.S., Li M., Mao S.X. (2003). Material-length-scale-controlled nanoindentation size effects due to strain-gradient plasticity. Acta Mater..

[10] Swadener J.G., Misra A., Hoagland R.G., Nastasi M. (2002). A mechanistic description of combined hardening and size effects. Scr. Mater..

[11] Yuan Z., Li F., Chen B., Xue F. (2014). The correlation between indentation hardness and material properties with considering size effect. J. Mater. Res..

[12] Voyiadjis G.Z., Yaghoobi M. (2017). Review of nanoindentation size effect: Experiments and atomistic simulation. Crystals.

[13] Ruiz-Moreno A., Hähner P. (2018). Indentation size effects of ferritic/martensitic steels: A comparative experimental and modelling study. Mater. Design.

[14] Durst K., Backes B., Franke O., Göken M. (2006). Indentation size effect in metallic materials: Modeling strength from pop-in to macroscopic hardness using geometrically necessary dislocations. Acta Mater..

[15] Pharr G.M., Herbert E.G., Gao Y. (2010). The indentation size effect: A critical examination of experimental observations and mechanistic interpretations. Annu. Rev. Mater. Res..

[16] Gale J.D., Achuthan A. (2014). The effect of work-hardening and pile-up on nanoindentation measurements. J. Mater. Sci.

[17] Joslin D.L., Oliver W.C. (1990). A new method for analyzing data from continuous depth-sensing microindentation tests. J. Mater. Res..

[18] Aldrich-Smith G., Jennett N.M., Hangen U. (2005). Direct measurement of nanoindentation area function by metrological AFM. Zeitschrift Metallkunde.

[19] Menčík J. (2012). Uncertainties and Errors in Nanoindentation. Nanoindentation Mater. Sci..

[20] Moharrami N., Bull S.J. (2014). A comparison of nanoindentation pile-up in bulk materials and thin films. Thin Solid Films.

[21] Hosemann P., Shin C., Kiener D. (2015). Small scale mechanical testing of irradiated materials. J. Mater. Res..

[22] Hardie C.D., Roberts S.G., Bushby A.J. (2015). Understanding the effects of ion irradiation using nanoindentation techniques. J. Nucl. Mater..

[23] Heintze C., Bergner F., Akhmadaliev S., Alstadt E. (2016). Ion irradiation combined with nanoindentation as a screening test procedure for irradiation hardening. J. Nucl. Mater..

[24] Chang C., Garrido M.A., Ruiz-Hervias J., Rodriguez J. (2017). On the possibility of reducing the pile-up effect on the Berkovich instrumented indentation tests. Int. J. Mech. Sci..

[25] Beck C.E., Hofman F., Eliason J.K., Maznev A.A., Nelson K.A., Armstrong D.E.J. (2017). Correcting for contact area changes in nanoindentation using surface acoustic waves. Scr. Mater..

[26] Hou X.D., Jennett N.M. (2017). A method to separate and quantify the effects of indentation size, residual stress and plastic damage when mapping properties using instrumented indentation. J. Phys. D Appl. Phys..

[27] Fischer-Cripps A.C. (2004). Nanoindentation.

[28] Chudoba T., Jennett N.M. (2008). Higher accuracy analysis of instrumented indentation data obtained with pointed indenters. J. Phys. D Appl. Phys..

[29] Hall E.O. (1951). The deformation and ageing of mild steel: III Discussion of results. Proc. Phys. Soc. B.

[30] Petch N.J. (1953). The cleavage strength of polycrystals. J. Iron Steel Inst..

[31] Nix W.D., Gao H. (1998). Indentation size effects in crystalline materials: A law for strain gradient plasticity. J. Mech. Phys. Solids.

